# Assessing the Nature of the Distribution of Localised States in Bulk GaAsBi

**DOI:** 10.1038/s41598-018-24696-2

**Published:** 2018-04-24

**Authors:** Tom Wilson, Nicholas P. Hylton, Yukihiro Harada, Phoebe Pearce, Diego Alonso-Álvarez, Alex Mellor, Robert D. Richards, John P. R. David, Nicholas J. Ekins-Daukes

**Affiliations:** 1 The Blackett Laboratory, Imperial College London, SW7 2AZ London, United Kingdom; 2 Department of Electrical and Electronic Engineering, Graduate School of Engineering, Kobe University, 1-1 Rokkodai, Nada Kobe, 657-8501 Japan; 3 Department of Electronic and Electrical Engineering, University of Sheffield, Sheffield, S1 3JD United Kingdom; 4 School of Photovoltaic and Renewable Energy Engineering, University of New South Wales, Sydney, Australia

## Abstract

A comprehensive assessment of the nature of the distribution of sub band-gap energy states in bulk GaAsBi is presented using power and temperature dependent photoluminescence spectroscopy. The observation of a characteristic red-blue-red shift in the peak luminescence energy indicates the presence of short-range alloy disorder in the material. A decrease in the carrier localisation energy demonstrates the strong excitation power dependence of localised state behaviour and is attributed to the filling of energy states furthest from the valence band edge. Analysis of the photoluminescence lineshape at low temperature presents strong evidence for a Gaussian distribution of localised states that extends from the valence band edge. Furthermore, a rate model is employed to understand the non-uniform thermal quenching of the photoluminescence and indicates the presence of two Gaussian-like distributions making up the density of localised states. These components are attributed to the presence of microscopic fluctuations in Bi content, due to short-range alloy disorder across the GaAsBi layer, and the formation of Bi related point defects, resulting from low temperature growth.

## Introduction

The incorporation of dilute quatities of bismuth into GaAs has been highlighted as a promising material system for a range of optoelectronic device applications, including telecommunications band (1550–1300 nm) lasers^1–3^, mid-IR photosensitive detectors^4–7^ and multi-junction photovoltaics (PV)^8–10^. The application of dilute bismide alloys to multi-junction PV could offer a suitable pathway to realising a highly desirable 1.0 eV band-gap sub-cell, which is crucial for the development of future four junction solar cells. Such a device has the potential to achieve power conversion efficiencies in excess of 50% under concentrated illumination^11^, an important milestone for making terrestrial concentrator PV systems a cost effective alternative to flat panel Silicon in the global PV market^12^.

A substantial reduction in band-gap energy is observed with increasing Bi incorporation, with reports of 80–90 meV/Bi% for Bi fractions in excess of 6%^13,14^. This large band-gap bowing is mainly the result of a prominent valence band anti-crossing interaction between resonant Bi energy levels and the GaAs host matrix, which results in a non-linear upward shift of the valence band edge^15^. Whilst the proximity of the Bi induced energy levels close to the valence band edge strongly affects its electronic structure, it has also been shown that the incorporation of Bi also modifies the conduction band^16^, with a linear downward shift of 33 meV/%Bi observed in GaAsBi quantum wells containing up to 5.9% Bi^17^.

Early studies of GaAsBi growth by metalorganic vapour phase epitaxy (MOVPE) were reported by^18,19^. More recently, GaAsBi with Bi fractions in excess of 10% and 7% has been grown successfully by molecular beam epitaxy (MBE)^20,21^ and MOVPE^22^, respectively. In all instances a low substrate temperature of <400 °C is required, with a low As_2_:Ga flux ratio of around 0.5^21^ favourable to achieving Bi incorporation in excess of 5%. The necessity for GaAsBi to be grown at low temperatures is mainly due to a large miscibility gap, which results in a poor solubility of Bi into the GaAs host lattice for all but a narrow range of growth temperatures^23^. This is attributed to the large atomic size difference between As and Bi atoms, leading to an increase in substitutional energy due to increased compressive strain^24^.

The large difference in atomic size between As and the substitutional Bi atom produces a number of interesting structural and optical properties in GaAsBi. The interaction of light-hole band edge states with Bi energy levels has been shown to introduce the presence of an inherent short-range alloy disorder as a result of the unconventional growth conditions^25^. Short-range alloy disorder manifests itself as microscopic fluctuations in Bi content across the growth layer, which result in non-uniform perturbations to the periodic potential of the lattice. This leads to the formation of a distribution of localised energy states that extend into the band-gap below some cut-off energy, often referred to as the mobility edge.

The presence of short-range alloy disorder in GaAsBi has often been identified, most commonly as a characteristic red-blue-red shift (or s-shape) evident in temperature dependent photoluminescence studies^26–31^. Recently, optical studies have sought to characterise the nature of the distribution of localised energy states in GaAsBi. Gogineni *et al*. have reported luminescence spectra that indicate the presence of an Urbach tail extending into the band-gap, suggesting that the localised states take an exponential distribution^32^. On the other hand, Imhof *et al*. have shown that modelling the distribution of localised states with both exponential and Gaussian components on two energy scales best reproduces the luminescence characteristics of GaAsBi^27^. This interpretation is also supported by Shakfa *et al*., who indicate that a two-component density of states made up of combinations of both exponential and Gaussian distributions can also describe well the luminescence quenching behaviour of GaAsBi^33^. In this paper, we seek to consolidate the work in the literature by applying a range of analytic techniques to temperature dependent photoluminescence measurements under varying excitation conditions, offering a consistent and comprehensive assessment of the nature of localised energy states in GaAsBi.

## Experimental Details

### Sample Growth

**Figure 1:**
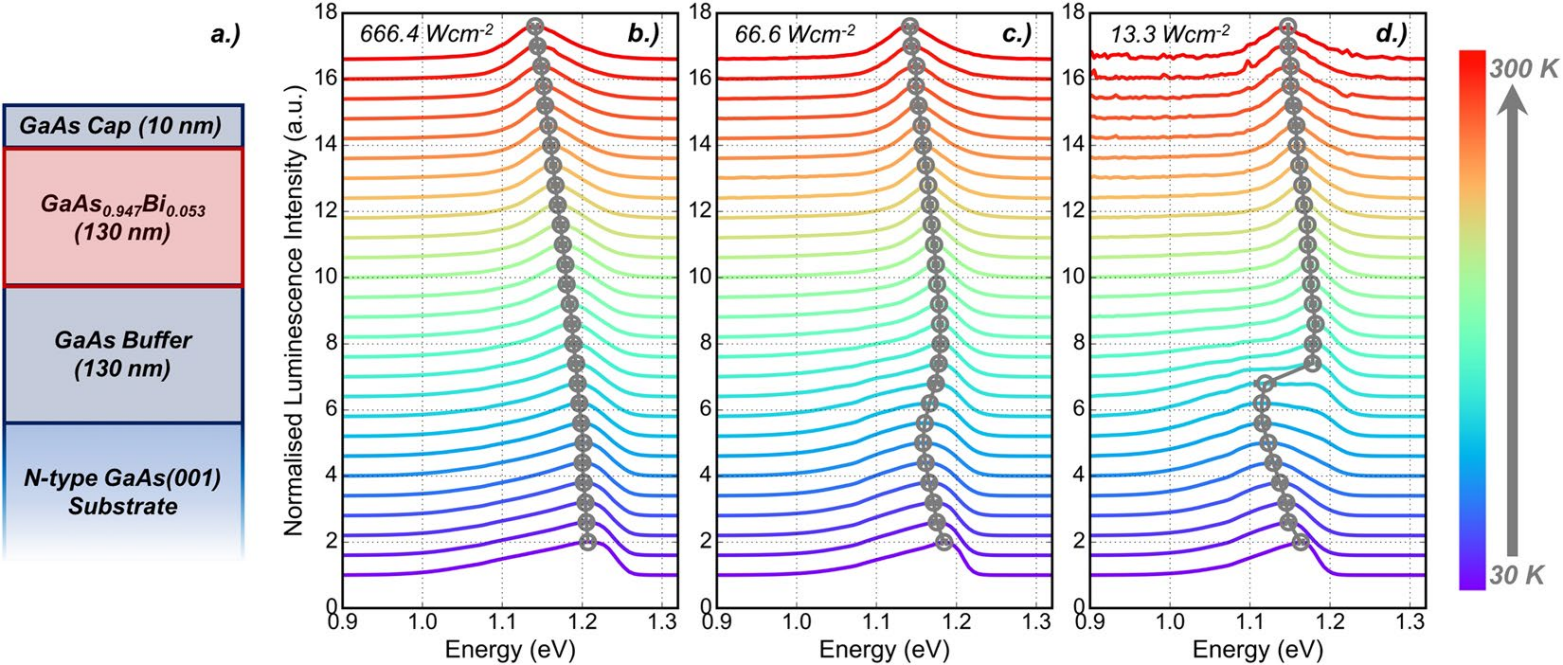
**(a)** Schematic diagram of the bulk GaAsBi sample studied in this work, grown via MBE. Representative temperature dependent PL spectra measured at **(b)** 666.4 *Wcm*^−2^ **(c)** 66.6 *Wcm*^−2^ and **(d)** 13.3 *Wcm*^−2^. The normalised luminescence intensity is offset for clarity. The peak luminescence energy is marked with grey symbols and the solid line is to guide the eye.

A bulk GaAsBi sample, with a targeted Bi fraction of 5.3%, was grown using an Omicron MBE - scanning tunnelling microscope system. A schematic diagram of the layer structure of the sample is shown in Fig. 1(a). Growth was undertaken on an n-type GaAs(001) substrate, which was outgassed at 450 °C for 30 minutes before being raised to 600 °C under an As_2_ flux for 30 minutes to desorb the native oxide on the substrate surface. An undoped GaAs buffer of thickness 130 nm was deposited at 570 °C using As_2_ in order to provide an atomically flat surface for subsequent epilayer growth. The sample was cooled to 340 °C and exposed to a Bi flux for 20 s, followed by a 30 s rest period in order to populate the Bi surface layer^34^. A 130 nm GaAsBi layer was deposited using an As_4_:Ga 2:1 atomic flux ratio to aid Bi incorporation^35^. Following deposition of the GaAsBi layer, a 10 nm GaAs cap was deposited under the same conditions as those used during deposition of the GaAsBi layer. A constant growth rate of 0.127 *μmh*^−1^, calibrated using reflection high-energy electron diffraction, was used across the entire sample. More detailed information on the recent growth of GaAsBi in this system is reported elsewhere^36^.

### Photoluminescence Characterisation

For the photoluminescence (PL) characterisation in this work the sample was mounted inside a closed-cycle helium cryostat, the temperature of which is controlled using an Oxford Instruments programmable temperature controller. It has been identified that Bi has a tendency to incorporate inhomogeneously during growth, with recent structural characterisations of the material showing the content of Bi decreasing exponentially after the first 10–25 nm of growth to a near constant level in the remainder of the layer^37,38^. With this in mind, to ensure that emission originates from the homogeneous region of GaAsBi closest to the surface, the sample was selectively excited using a continuous wave Power Technology Inc. diode laser emitting at 3.06 eV, with incident excitation power ranging from 0.33–1494 *Wcm*^−2^. The luminescence was dispersed through a 0.5 m Princeton Acton SP2500i spectrometer, with a 300 lines/mm grating blazed at 1 *μm*. A Hamamatsu near-infrared PMT was used to collect the sample luminescence and the signal measured with standard phase sensitive detection techniques using a Stanford SR830 lock-in amplifier.

## Presentation and Discussion of Results

### Temperature Dependent Photoluminescence

**Figure 2:**
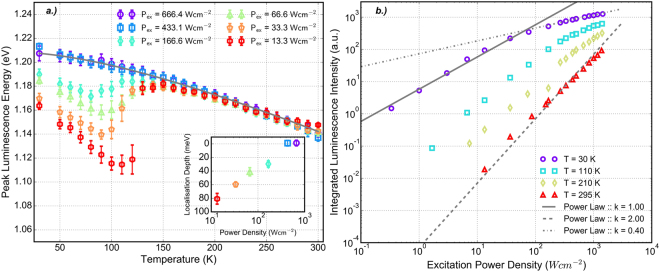
**(a)** PL peak energy position as a function of temperature for varying incident excitation power. The extracted localisation energy is plotted inset. **(b)** Excitation power dependence of the integrated luminescence intensity at various temperatures. The solid, dashed and dot-dashed lines indicate power law relations with exponents *k* = 1.0, *k* = 2.0 and *k* = 0.4 respectively.

A representative plot of the measured temperature dependent PL spectra is shown in Fig. 1 for (b) 666.4 *Wcm*^−2^ (c) 66.6 *Wcm*^−2^ and (d) 13.3 *Wcm*^−2^. The peak luminescence energy as a function of temperature is shown in Fig. 2(a) for incident excitation power ranging from 13.3–666.4 *Wcm*^−2^. The error bars on each data point represent the statistical uncertainty associated with calculating the average PL peak position.

The presence of a characteristic red-blue-red shift in the peak luminescence energy is clearly observed in Fig. 2(a), and the origin of this behaviour can be explained by considering the carrier distribution amongst the localised density of states (LDOS) at different temperatures^39^. At low temperature, both deep and shallow localised states are occupied by electron-hole pairs. Due to the low temperature, carriers have a small thermal energy and remain trapped until they are able to recombine. As the temperature increases electron-hole pairs in the shallow energy states close to the valence band edge gain enough thermal energy to escape and become trapped by deeper level localised states. This behaviour results in a rapid red shift in the PL peak energy and can be seen in Fig. 2(a) between 30–90 K. As carriers gain greater thermal energy however, they are able to complete multiple hops between states, allowing carriers to escape the deepest traps and occupy a range of shallower energy states. This leads to a blue shift in peak emission energy between 90–140 K. Above 150 K, the PL peak energy follows a further red shift as the carriers have enough thermal energy to distribute themselves randomly amongst the large density of shallow localized states in the vicinity of the mobility edge.

It is clear from Fig. 2(a) that the magnitude of the red-blue-red shift at low temperature is highly dependent on the incident excitation power. Under excitation at 13.3 *Wcm*^−2^ the low carrier density allows emission from a wide range of localised states leading to an extended red shift up to 100 K. However, as the incident power density is increased, the larger density of photogenerated carriers begins to fill the localised states furthest from the band-edge resulting in a decrease in the magnitude of the red-blue-red shift at low temperature. The temperature dependence of the luminescence peak energy under excitation at 666.4 *Wcm*^−2^ shows no evidence of the characteristic s-shape observed under lower power conditions, and is well approximated by the empirical Varshni relation, which describes the temperature induced expansion of the lattice^40^.

1 $${E}_{gap}(T)={E}_{gap}(0)-\frac{\alpha {T}^{2}}{T+\beta }$$

With Varshni parameters *α* = 0.42 *meVK*^−1^ and *β* = 270 *K*, which sit comfortably in the range of previously reported parameters for GaAsBi where *α* = 0.1–0.63 *meVK*^−1^ and *β* = 120–295 *K*^26,30,31,41,42^. The close agreement with the Varshni relation suggests that the majority of tail states in the distribution are filled under 666.4 *Wcm*^−2^ excitation and that the emission characteristics are dominated by the much larger density of shallow states close to the mobility edge. In the higher temperature regime, above 200 K, the data tends towards a common reduction in band-gap energy that is described well by the Varshni relation. In this regime the thermal energy of carriers is enough to allow a significant number to be excited from the LDOS to the mobility edge where they are described as de-localised. If the Varshni fit is taken to represent the position of the mobility edge at low temperature, an estimate of the depth of the localised states in the band-gap can be made by calculating the difference between this and the minimum of the red-blue shift. The inset graph in Fig. 2(a) shows a decrease in the localisation energy from 80 ± 8 *meV* at 13.3 *Wcm*^−2^ to 29 ± 5 *meV* at 166.6 *Wcm*^−2^ before the difference becomes negligible due to the filling of localised states at higher powers. This further illustrates the strong dependence of localised carrier behaviour on the incident excitation power.

### Power Dependent Photoluminescence

The integrated spectral intensity of the luminescence peak is depicted as a function of increasing excitation power in Fig. 2(b), presented at a number of different temperature points. The integrated PL intensity has been shown to be proportional to the incident excitation power with exponent, *k*^43^;

2 $${I}_{PL}\propto {P}_{ex}^{k}$$

The value for *k* varies depending on the nature of radiative recombination taking place. Schmidt *et al*. show^43^ that for bimolecular recombination processes, where the electron and hole concentrations can be considered approximately equal, the exponent *k* tends towards 2.0. In the case where the concentrations of electrons and holes are different, i.e. if emission is dominated from a considerably smaller density of localised energy states, the recombination process is described as monomolecular and the exponent *k* tends towards 1.0.

This power law analysis is applied to the integrated luminescence intensity measured at 30 K and at 300 K and is shown in Fig. 2(b) as the dashed and dot-dashed lines respectively. A good agreement between the power law with exponent *k* = 2.0 and the data at 300 K is observed, indicating that the dominant radiative recombination process is bimolecular. At this temperature carriers have enough thermal energy to escape the LDOS and distribute themselves at the mobility edge. As the effective concentrations of electrons and holes can be considered similar under these conditions a bimolecular process is observed. At 30 K a power law with exponent *k* = 1.0 produces a good fit to the data below 100 *Wcm*^−2^, indicating that a monomolecular radiative recombination process is dominant at low temperature. This is consistent with emission being dominated by recombination from carriers frozen in localised states, where the density of states is likely to be much smaller than that in the continuum above the mobility edge. The experimental data is observed to deviate above 100 *Wcm*^−2^ from the solid grey line and follows a power law with exponent *k* = 0.4, indicated by the dot-dashed line. This saturation of the PL intensity at high excitation power is attributed to an increase in non-radiative recombination amongst shallow localised states resulting from local heating of the sample. The filling of deeper localised states at high excitation powers means localised excitons will occupy states close to the mobility edge. The smaller energy separation between these states, in conjunction with increased thermal energy due to local heating, will increase the probability of carriers being thermally activated out of the localised states and able to reach non-radiative centers caused by growth related impurities or Bi-induced defects. This effect is most prominently observed at lower temperatures (30 K and 110 K in Fig. 2(b)) as the average thermal energy of carriers increases at higher temperatures. This results in a higher probability of non-radiative recombination under all excitation conditions, which is reflected by the overall quenching of the PL intensity as the measurement temperature is increased.

It has been reported that the distribution of mid-gap states resulting from alloy disorder commonly take the form of an exponential tail^44–46^, which has previously been observed in GaAsBi alloys^32,47^. On the other hand, there is evidence that suggests a Gaussian distribution of localised states extending from the mobility edge may better describe the LDOS in GaAsBi^27,33^. Given that the luminescence lineshape reflects the profile of the density of states in the vicinity of the band-edge, its nature can be investigated by examining the PL spectra under different excitation conditions^48,49^. If at a certain generation rate it is assumed that the emission is dominated by excitons recombining from energy levels up to some maximum, *E*_*Max*_, of the LDOS distribution, and that emission at each of these energy levels can be described with a Gaussian profile, the effective luminescence intensity can be described by;

3 $${I}_{eff}(E)={\int }_{-\infty }^{{E}_{Max}}{g}_{Gauss}(E,{E}_{0},{\sigma }_{Gauss})\times {g}_{LDOS}({E}_{0},{E}_{Mob},{\sigma }_{LDOS})\cdot d{E}_{0}$$

where *E*_0_ defines the centre energy of emission from each energy state in the LDOS distribution up to *E*_*Max*_ and *E*_*Mob*_ defines the position of the mobility edge, below which the density of states is described by *g*_*LDOS*_. It is also assumed that the rate of radiative recombination is defined by the density of states at each energy level^48^. The profile of the LDOS, defined by *g*_*LDOS*_, can take the form of either a Gaussian or Exponential distribution given by;

4 $${g}_{Gauss}(E,{E}_{0},{\sigma }_{Gauss})=\frac{1}{\sqrt{2\pi {\sigma }_{Gauss}^{2}}}\,\exp \,(-\frac{{(E-{E}_{0})}^{2}}{2{\sigma }_{Gauss}^{2}})$$

5 $${g}_{Exp}(E,{E}_{0},{\sigma }_{Exp})=\frac{1}{\sqrt{{\sigma }_{Exp}}}\,\exp \,(-\frac{(E-{E}_{0})}{{\sigma }_{Exp}})$$

**Figure 3:**
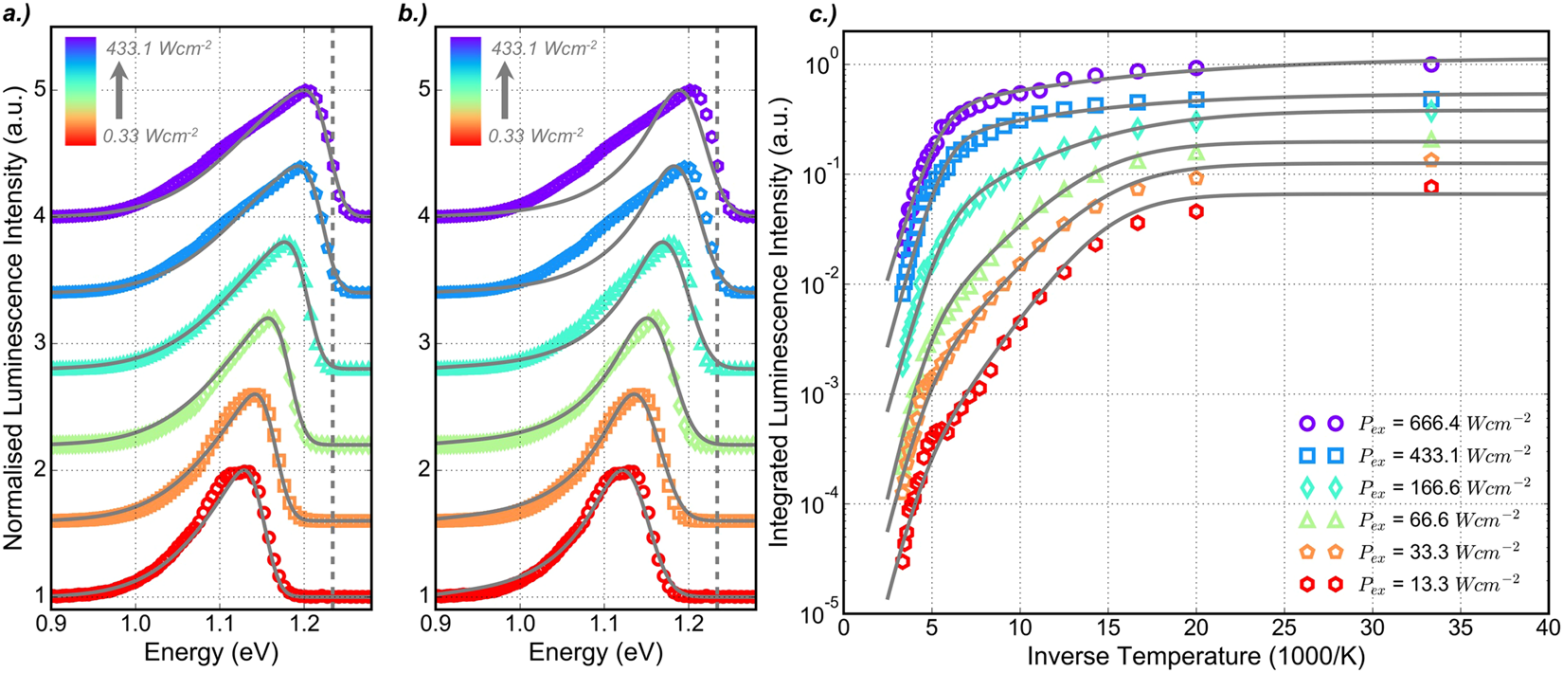
Normalised luminescence intensity spectra (offset for clarity) measured at 30 K as a function of excitation intensity. The solid lines indicate best fits using Equation 3 and the parameters listed in Table 1 for **(a)** *g*_*LDOS*_ = *g*_*Gauss*_ and **(b)** *g*_*LDOS*_ = *g*_*Exp*_. The dashed line indicates the position of the mobility edge, *E*_*Mob*_. **(c)** Integrated luminescence intensity as a function of inverse temperature for varying incident excitation power. The solid curves are produced using Equation 6 with activation energies summarised in Table 2.

Here, *σ*_*Gauss*,*Exp*_ denotes the standard deviation of the Gaussian and Exponential distributions respectively. Normalised photoluminescence spectra measured at 30 K for a range of excitation powers are plotted in Fig. 3(a) and (b), offset for clarity. Lineshape analysis is carried out at 30 K due the effects of carrier localisation and state filling being most pronounced at low temperature. Incident excitation powers ranging from 0.33–433.1 *Wcm*^−2^ are considered as the vanishing s-shape, evident in Fig. 2(a), for higher powers indicates a saturation of the deeper energy states in the LDOS distribution. Both Gaussian (Fig. 3(a) and Exponential (Fig. 3(b)) LDOS distributions are considered, with the associated fitting parameters summarised in Table 1. The standard deviation parameters describing both *g*_*Gauss*_ and *g*_*LDOS*_ are determined for *P*_*Ex*_ = 0.33 *Wcm*^−2^, as this condition best reflects the profile of the LDOS, and are kept fixed as the excitation power is increased. The luminescence lineshape is therefore governed by the position of *E*_*Max*_, which is represented in Table 1 as the localisation energy defined as *E*_*Loc*_ = *E*_*Mob*_ − *E*_*Max*_, where *E*_*Mob*_ = 1.233 *eV* extracted from the model fit to the data under the lowest excitation power. The normalised mean average error (NMAE), which defines the average absolute uncertainty between the experimental data and that predicted by the lineshape model at each energy, is used as the metric for determining the suitability of each LDOS distribution.

**Table 1:** Summary of parameters used to fit the data in Fig. 3(a) and (b).

	*g*_*LDOS*_ = *g*_*Gauss*_		*g*_*LDOS*_ = *g*_*Exp*_	
*P*_*Ex*_ (*Wcm*^−2^)	*E*_*Loc*_(*meV*)	NMAE (%)	*E*_*Loc*_(*meV*)	NMAE (%)
0.33	80.47	0.62	84.99	0.71
3.00	66.01	0.50	71.61	0.78
13.33	50.60	0.70	56.32	0.87
76.63	28.44	0.42	37.20	1.33
266.54	10.14	0.63	23.82	2.01
433.13	2.43	0.79	18.09	2.42

Whilst the exponential distribution produces a close fit to the experimental data in Fig. 3(b) for *P*_*Ex*_ = 0.33 *Wcm*^−2^, with *σ*_*LDOS*_ = 63 *meV* and *σ*_*Gauss*_ = 22 *meV*, it is unable to reproduce the data for *P*_*Ex*_ = 433.1 *Wcm*^−2^. The exponential profile underestimates the lineshape below *E*_*Mob*_ and this is reflected by a maximum NMAE of 2.42%. However the Gaussian distribution fit depicted in 3 (a), with *σ*_*LDOS*_ = 100 *meV* and *σ*_*Gauss*_ = 14.5*meV*, is able to reproduce the experimental data well for all excitation powers, with a maximum recorded NMAE of 0.788%. The luminescence lineshape is reproduced well by increasing *E*_*Max*_ with incident excitation power, resulting in a decrease in *E*_*Loc*_ that indicates the filling of localised states with increased photogenerated carrier density. The values of *E*_*Loc*_ reported in Table 1 are in good agreement with the decreasing localisation energy observed in the inset graph of Fig. 2(a). It can therefore be concluded that a Gaussian profile is a good first description of the LDOS distribution in this GaAsBi sample, where the larger density of energy states close to the mobility edge may be attributed to the proximity of Bi energy levels close to the valence band maximum.

### Thermal Quenching of Photoluminescence

A plot of the integrated luminescence intensity as a function of inverse temperature is shown in Fig. 3(c) for a range of incident excitation powers. The Arrhenius equation with two activation energies, which describe the thermal activation of carriers in two temperature regimes, is found to best describe the data. It is given by;

6 $${I}_{Int}(T)=\frac{{I}_{0}}{1+{C}_{1}\,{\exp }\,(-{E}_{a1}/{k}_{B}T)+{C}_{2}\,{\exp }\,(-{E}_{a2}/{k}_{B}T)}$$

where *I*_0_ is the integrated intensity at 30 K, and *C*_1_ and *C*_2_ are fitting parameters relating to *E*_*a*1_ and *E*_*a*2_ respectively. Two temperature regimes in the thermal quenching of the luminescence are considered. *E*_*a*1_ describes the thermal activation of carriers in the high temperature regime (3.3 to 5 1000/K), which, according to Fig. 2(a), corresponds to the temperature range where carriers can be considered as de-localised from the LDOS (200 to 300 K). *E*_*a*2_ describes the thermal activation of carriers at lower temperatures (12.5 to 5.9 1000/K), which corresponds to the temperature range where carriers are able to hop between localised energy states in the LDOS (80 to 170 K). By linearising Equation 6 and fitting the data in the two temperature regimes, the activation energies are extracted and listed in Table 2.

**Table 2:** Summary of activation energies used to fit the data in Fig. 3(c) with Equation 6.

*P*_*Ex*_ (*Wcm*^−2^)	*E*_*a*1_(*meV*)	*E*_*a*2_(*meV*)
13.3	130 ± 8	44 ± 1
33.3	127 ± 8	36 ± 1
66.6	138 ± 9	34 ± 2
166.6	115 ± 7	21 ± 1
433.1	118 ± 6	13 ± 1
666.4	105 ± 7	10.2 ± 0.7

The fitted curves are illustrated in Fig. 3(c) by the solid grey lines and a good fit to the experimental data is observed for all excitation powers. The fitted values of *E*_*a*1_ show little evidence of a trend between high and low excitation power, however *E*_*a*2_ is clearly observed to decrease with increasing excitation power. This is attributed to the filling of localised states as the photo-generated carrier density increases. The values of *E*_*a*1_ are comparable with the standard deviation *σ*_*LDOS*_ = 100 *meV* of the Gaussian *g*_*LDOS*_ used to reproduce the power dependence of the PL lineshape at 30 K in Fig. 3(a). This activation energy is therefore attributed to the thermal de-localisation of carriers from the LDOS to the mobility edge. The difference in behaviour and magnitude of the two activation energies suggests that they describe processes originating from different parts of the LDOS and that the single Gaussian profile may not be sufficient to fully describe the nature of the localised states in GaAsBi.

**Figure 4:**
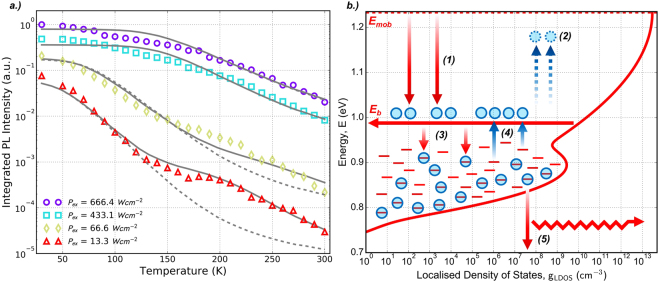
**(a)** The thermal quenching of integrated luminescence intensity for varying incident excitation power. The grey curves illustrate modelled data obtained by solving the rate model described by Equations 9, 10 and 11 considering a uniform (dashed) and two-component (solid) LDOS. **(b)** Schematic diagram of a dual-component LDOS depicting the processes described in the rate model.

Figure 3(c) illustrates evidence of a plateau in the luminescence quenching, which is most obvious for *P*_*Ex*_ = 13.3 *Wcm*^−2^ and *P*_*Ex*_ = 33.3 *Wcm*^−2^, which is not well reproduced by the Arrhenius equation. This plateau is more clearly observed in a plot of integrated luminescence intensity as a function of temperature as illustrated in Fig. 4(a). Shakfa *et al*. suggest that this results from a two-component LDOS distribution, where at intermediate temperatures the radiative recombination of localised excitons from a deeper distribution of states balances the increasing non-radiative recombination as carriers in shallow states are excited above the mobility edge^50^. Evidence of a the dual-component distribution to the localised states is also observed by Imhof *et al*., who show excellent agreement with experimental data using a kinetic Monte Carlo model taking into account exponential and Gaussian distributions on two energy scales to describe the hopping dynamics of carriers amongst the localised states^27^.

To describe the power dependence of the luminescence thermal quenching a rate model is proposed that will explicitly take into account the filling of localised states^28,51^. It is considered that carriers are able to radiatively recombine from the LDOS that exists within the band-gap. The distribution can be made up of both single and double Gaussian components, following the observation for GaAsBi in^27^, and is given by;

7 $${g}_{LDOS}={N}_{0}[(1-y)\cdot {g}_{Gauss}(E,{E}_{Mob},{\sigma }_{1})+y\cdot {g}_{Gauss}(E,{E}_{Off},{\sigma }_{2})]$$

where *E*_*Off*_ is the energy offset of the second Gaussian component from the mobility edge, *E*_*Mob*_, and y describes its fractional contribution to *g*_*LDOS*_. *N*_0_ describes the density of localised states at the mobility edge. The total carrier density in the LDOS is defined as *n*(*E*) = *n*_*f*_(*E*) + *n*_*e*_(*E*), where *n*_*f*_(*E*) and *n*_*e*_(*E*) denote filled and empty localised states respectively. A schematic diagram of the energy levels in the LDOS as a function of the density of states is shown in Fig. 4(b). Empty localised states are represented by the short red lines and the filled states are populated with localised excitons (blue circles). Photo-generated carriers are injected into the LDOS at a constant rate *n*_*gen*_ (process (1) in Fig. 4(b)), where they relax to occupy the energy *E*_*b*_ with carrier density *n*_*b*_. *E*_*b*_ is defined as an energetic barrier, below which localised excitons can either recombine radiatively with rate *k*_*r*_ (process (5)) or become thermally activated out of a localised state to join *n*_*b*_, where they are able to transfer to other localised states (process (3)) or recombine non-radiatively with rate *k*_*nr*_ (process (2)). The probability of escape from a localised state at energy E to *E*_*b*_ is given by;

8 $$\beta (E)={\beta }_{0}\,{\exp }\,[-({E}_{b}-E)/{k}_{B}T]$$

where *β*_0_ describes the intrinsic rate of thermal activation at low temperature. The effect of state filling in the LDOS implies that the capture and thermal activation processes are proportional to the number of empty localised states^51^. The pair of coupled rate equations describing this system are defined;

9 $$\frac{d{n}_{b}}{dt}={n}_{gen}-{n}_{b}{k}_{nr}-\int {n}_{b}{k}_{c}{n}_{e}(E)\cdot dE+\int {n}_{f}(E)\beta (E)\cdot dE$$

10 $$\frac{d{n}_{f}(E)}{dt}=-\,{n}_{f}(E)\beta (E)-\,{n}_{f}(E){k}_{r}+{n}_{b}{k}_{c}{n}_{e}(E)$$

where Equation 9 describes the rate of change of carriers at energy *E*_*b*_, with terms on the right hand side of the equation describing, from left to right, the processes in Fig. 4(b) (1) the density of photo-generated carriers; (2) non-radiative recombination at *E*_*b*_ with rate *k*_*nr*_; (3) the total number of carriers captured into empty localised states from *n*_*b*_ with capture coefficient *k*_*c*_; and (4) the total number of carriers thermally activated from the LDOS to *E*_*b*_. The coupled Equation 10 describes the rate of change of carriers occupying localised energy states in the LDOS with terms on the right hand side, from left to right, describing processes in Fig. 4(b) where carriers; (4) are thermally activated to *E*_*b*_; (5) recombine radiatively with rate *k*_*r*_; and (3) are captured into empty localised states. The rate of radiative recombination from the LDOS is assumed to remain constant at all temperatures whilst the rate of non-radiative recombination increases with temperature as ${k}_{nr}={k}_{n{r}_{0}}\cdot [1-\,{\exp }\,(-\,T/20)]$^51^, which saturates at around 150 K in agreement with the common de-localisation of carriers observed in Fig. 2(a). Under steady state conditions *dn*_*b*_/*dt* = *dn*_*f*_/*dt* = 0 and Equations 9 and 10 are solved numerically for *n*_*b*_. Finally, the integrated luminescence intensity is calculated from the proportion of filled localised states that recombine radiatively;

11 $${I}_{Int}=F\int {k}_{R}{n}_{f}(E)\cdot dE$$

where *F* is a normalisation constant. The calculated quenching of luminescence using the rate model and Equation 11 are plotted in Fig. 4(a). A reasonable fit to the data is achieved using the rate model parameters *k*_*r*_ = 1 × 10^9^ *s*^−1^, ${k}_{n{r}_{0}}=4\times {10}^{15}\,{s}^{-1}$, *k*_*c*_ = 7 × 10^6^ *cm*^2^*s*^−1^ and *β*_0_ = 4 × 10^14^ *s*^−1^, which are in good agreement with the range of values reported in^28,51^ for dilute-nitride based quantum wells and GaAsBi alloys up to 6.7% respectively. The LDOS is made up of two Gaussian distributions, one extending from the mobility edge and a second off-set deeper into the band-gap. The profile of *g*_*LDOS*_ is determined from the fit to the data under the lowest excitation power (*P*_*Ex*_ = 13.3 *Wcm*^−2^), which best reflects emission from the LDOS. A good fit is observed using the parameters *N*_0_ = 3 × 10^13^ *cm*^−3^, *E*_*Off*_ = 340 *meV*, *σ*_1_ = 62 *meV*, *σ*_2_ = 20 *meV* and *y* = 0.003%. *E*_*Mob*_ is assumed to take the value of the de-localised band-edge and is calculated at each temperature using Equation 1 with parameters discussed earlier in this paper. The energy scales of the two Gaussian distributions are in good agreement with those extracted from the kinetic Monte Carlo model reported in^27^ (where the energy scales attributed to alloy disorder and Bi atom clustering are given by *ε*_1_ = 45 *meV* and *ε*_2_ = 11 *meV* respectively). Although the contribution of the second Gaussian component appears very small at *y* = 0.003% it is essential to reproducing the plateau in the PL quenching, which is attributed to competing radiative and non-radiative processes in the offset Gaussian density of states^50^. The dashed lines in Fig. 4(a) indicate the results of the model calculated with *y* = 0% and it is clear that the fit does not agree well with the data, especially under low excitation powers. This suggests that the profile of the LDOS is more complex than the uniform Gaussian profile predicted from the lineshape analysis in Fig. 4(a), but consistent with the dual component prediction reported in^27,33^. The wider distribution extending from the mobility edge reflects the short-range alloy disorder caused by microscopic fluctuations in Bi content during growth. The origin of the deeper contribution to the LDOS is attributed to Bi induced point defects in the material, with the observed 340 meV offset in good agreement with hole traps observed in a range of bulk GaAsBi layers^52^.

Additional data sets in Fig. 4(a) are reproduced with the rate model for increasing excitation powers. To illustrate the consistency of the model, all rate parameters and those describing the magnitude and profile of the LDOS are fixed. The only parameters allowed to vary are those describing the energetic barrier *E*_*b*_ and the density of photo-generated carriers *n*_*Gen*_ and these are summarised in Table 3. As with the data at *P*_*Ex*_ = 13.3 *Wcm*^−2^, a good fit to the highest excitation power is observed by increasing the incident photo-generation rate by a factor of 13 and simultaneously decreasing the localisation energy, defined as the difference between the barrier energy and the position of the mobility edge, by 120 meV. This value of the localisation energy is comparable with the activation energies summarised in Table 2 describing the thermal delocalisation of carriers from the LDOS to the mobility edge. The increase in the barrier energy, and subsequent decrease in localisation depth, with increasing excitation power is a strong indication of localised state filling in the LDOS, which is consistent with the power dependent analysis of the peak luminescence energy and spectrum lineshape. The rate model here demonstrates that a uniform LDOS distribution is not sufficient to describe all aspects of the temperature dependent nature of the photoluminescence, with a two-component Gaussian profile able to best represent the thermal quenching of the luminescence at different incident excitation powers.

**Table 3:** Summary of power dependent rate model parameters.

*P*_*Ex*_ (*Wcm*^−2^)	*n*_*Gen*_(*cm*^−3^)	*E*_*Loc*_ = *E*_*Mob*_ − *E*_*b*_(*meV*)
13.3	1 × 10^19^/13	120
66.6	1 × 10^19^/4.3	85
433.1	1 × 10^19^/2.2	8
666.4	1 × 10^19^	0

## Conclusions

In summary, a comprehensive assessment of the nature of the localised distribution of energy states in GaAsBi is presented, applying a range of analytic techniques to temperature dependent photoluminescence measurements under different excitation power conditions. Strong evidence of carrier localisation is observed in the characteristic low temperature red-blue-red shift in PL peak energy, which is observed to decrease in magnitude with increasing excitation power indicating the filling of deep localised states. The localisation energy is estimated from the magnitude of the red-blue shift at low temperature and is shown to decrease from 80 ± 8 *meV* at 13.3 *Wcm*^−2^ to 29 ± 5 *meV* at 166.6 *Wcm*^−2^, illustrating the strong dependence of localised carrier behaviour on the incident excitation power.

The nature of the localised density of states is investigated by considering the evolution of the lineshape at low temperature. Both Exponential and Gaussian tail distributions are considered with the best fit to the experimental data observed assuming a Gaussian profile with standard deviation *σ*_*Gauss*_ = 100 *meV* and a localisation energy up to 81 *meV*. An Arrhenius analysis of the integrated luminescence intensity indicates the presence of two thermal activation regimes, where the activation energy describing the thermal delocalisation of carriers shows good agreement with the characteristic energy of the Gaussian distribution used to reproduce the photoluminescence lineshape.

A rate model describes the thermal quenching well assuming a dual-component LDOS made up of two Gaussian distributions with standard deviations *σ*_1_ = 62 *meV* and *σ*_2_ = 20 *meV*. The wider Gaussian is attributed to the effects of short-range alloy disorder whilst the narrow Gaussian, offset from the mobility edge by 340 *meV*, is attributed to excess hole traps caused by Bi-induced point defects during growth. The power dependence of the thermal quenching is also well reproduced, with the decreasing localisation energy from 120–0 *meV* indicating the filling of localised states under high excitation powers.

The conclusions drawn from the analytic techniques presented here are consistent with a dual component Gaussian distribution of localised states in GaAsBi. This furthers our understanding of the alloy and contributes to the continuing material development of disordered semiconductors, crucial for the next generation of optoelectronic devices.
